# Sex and gender in perioperative cardiovascular research: protocol for a scoping review

**DOI:** 10.1186/s13643-024-02716-9

**Published:** 2025-01-08

**Authors:** Ana Sjaus, Nicole Fakhory

**Affiliations:** 1 Department of Anesthesia, Pain Management and Perioperative Medicine, Dalhousie University, IWK Health Centre, 5850 University Avenue, Halifax, NS Canada; 2 Department of Women’s and Obstetric Anesthesia, IWK Health Centre, Halifax, NS Canada; 3 Faculty of Medicine, University of Ottawa, Ottawa, ON Canada

**Keywords:** Sex differences, Gender, Sex specific analysis, Perioperative complications, Anesthesia, Myocardial injury after non-cardiac surgery, Perioperative myocardial infarction, Cardiovascular complications, Perioperative stroke, Individualized medicine

## Abstract

**Background:**

The inadequate inclusion of sex and gender in medical research has resulted in biased clinical guidance and disparities in knowledge and patient outcomes. Despite efforts by regulatory and funding agencies, opportunities to generate sex-specific knowledge are frequently overlooked. While certain disciplines in cardiovascular medicine have made notable progress, these advances have yet to permeate the literature on perioperative cardiovascular complications in non-cardiac surgery.

Prompted by the recent findings on sex-specific perioperative cardiovascular outcomes, this review aims to scope the literature in this field and categorize methodological approaches used to incorporate sex and gender in studies of this patient population.

**Methods:**

Joanna Briggs Institute (JBI) methodology for scoping reviews will be followed in stages elaborated by Levac (2010). A comprehensive search strategy will be used to identify relevant primary research published since 2010. Screening will be performed by independent reviewers using predefined inclusion and exclusion criteria. Data will be extracted from full text and supplementary materials of selected articles. Results will be presented as proportions of studies reporting sex and gender, the assigned purpose of these variables in analysis, and where they are reported in the article. In addition, articles will be mapped to the source, country of origin, and year of publication. Narrative summaries will be provided to outline key findings and assess the depth of the literature within each of the major topics (risk assessment/prediction, diagnosis, treatment, prognosis, and outcomes).

**Discussion:**

Increasing recognition of the profound and complex implications of sex and gender in medicine has fuelled calls for greater attention to participation equity, sex-specific analysis and reporting. Focusing on perioperative cardiovascular complications, this review has the potential to identify knowledge gaps for future research, as well as areas of strength that could support formal knowledge synthesis or secondary analysis of data from past research.

**Scoping review registration:**

Submitted on August 15th, 2023 (Web of Science osf.io/u25sf)

## Introduction

Sex and gender have historically, for a variety of reasons, been overlooked in medical research [1]. For much of the last century, medical research - including the field of cardiovascular medicine - has been predominantly male centric, often excluding female and pregnant participants or failing to report on the now well-known differences between the biological sexes [2]. The resulting gaps in clinical knowledge and outcomes have been documented in various areas of medicine [3]. In cardiovascular medicine, the substantial improvements in care and outcomes among male, have not been equally realized in the female population [4–6]. Restrictive enrollment and failures to utilize appropriate methodologies have, ultimately, led to incomplete and poorly generalizable knowledge that created significant disparities. Biased clinical guidance and medical education are among the many knowledge translation implications that have perpetuated these gaps [2].

Over the last two decades, major funding and regulatory agencies have aimed, through a variety of guidelines and policies, to address these research disparities [7–9]. While there are signs that enrollment is increasingly inclusive, reporting on sex and gender remins inconsistent [10]. In cardiovascular research, however, examples of sex-specific reporting of differences across the age continuum reflect the growing recognition of the scientific imperative for sex-disaggregated analysis [4].

In 2019, citing the mounting evidence of male-female differences in pathophysiology, symptomatology, effectiveness of interventions, prognosis, and outcomes of cardiovascular diseases, the Canadian Cardiovascular Society endorsed the inclusion of sex and gender considerations in guideline development [11, 12]. In contrast, the field of perioperative cardiovascular medicine appears to have been, until very recently, slow to integrate these developments [13]. This lack of progress is reflected in the absence of sex-specific considerations in key North American guidelines for pre-operative cardiovascular evaluation and management, including those issued in 2017 by the Canadian Cardiovascular Society and in 2014 by the American College of Cardiology and American Heart Association (ACC/AHA). However, signaling a shift in position from “unaware” to “problem aware,” the more recent 2022 Guidelines from the European Society of Cardiologists, briefly acknowledge the lack of evidence for specific populations including “men and women” [14]. Published in 2021, PREECLAMPSIA-VISION examplifies this growing awareness. By re-analyzing the data from the VISION study that had previously established an association between myocardial injury following non-cardiac surgery (MINS) and 30-day mortality, it found that the self-reported history of preeclampsia in female participants was independently associated with an increased risk of MINS [15]. This example highlights the increased perioperative cardiovascular risks in women with the history of adverse pregnancy outcomes. Similarly, the recent work of Kwon et al. using a large South Korean database of over 30,000 surgical cases, found male-female differences in the incidence of MINS and subsequent long-term mortality [16].

While much of the recent discovery highlights gaps in the realm of women’s cardiovascular health, failing to appropriately account for sex differences can negatively impact both sexes [17]. Given that the perioperative period is a time of increased cardiovascular morbidity, evidence to support individualized risk assessments and a management plans is essential. Research that treats sex or gender in analysis as confounders or “nuisance” variables, assumes their effect to be the weighted average of sexes or genders [18]. Unless the analysis is stratified, or disaggregated, any interaction by sex or gender with the exposure of interest remains unexamined. The latter approach, however, requires larger overall sample sizes to ensure sufficient statistical power.

To provide truly personalized care, clinicians require sex- and gender-specific knowledge. This review is the first systematic investigation of primary research in perioperative cardiovascular medicine that addresses these variables. By outlining the methodological approaches to sex and gender inclusion, describing trends over time, and identifying the research gaps and opportunities for update, the findings of this review aim to inform clinical practice, guideline development, funding priorities and further research including knowledge synthesis. These overarching objectives align well with the goals of scoping review methodology.

## Methods

For the purpose of this review, we adopt the Canadian Institutes of Health Research (CIHR) definitions of sex and gender [19].

### Scoping review framework

This scoping review will be conducted following the Joanna Briggs Institute (JBI) methodology for scoping reviews as described by Arksey and O’Malley, in stages elaborated by Levac and updated by Peters in 2021 [20–22]. The Preferred Reporting Items for Systematic Reviews and Meta-Analysis extension for scoping reviews (PRISMA-ScR) will be used to guide the reporting and presentation of findings [23].

### Stage 1: identifying the research question

#### Research question

What proportion of primary research on perioperative cardiovascular complications in non-cardiac surgery has reported on sex and gender, and how are these variables used in analysis?

#### Objectives

The primary objective is to determine the frequency of and categorize the reporting of sex/gender in the body of research addressing perioperative cardiovascular complications in non-cardiac surgery, map to the source, geographic origin, authors’ affiliation, specialty, methodology, methodological purpose of sex/gender and the date of publication.

Secondary objectives include presenting the frequency of reporting and narrative summaries of the findings of research within each major thematic area: epidemiology, symptomatology, risk prediction and stratification, prevention, treatment, and outcomes.

### Stage 2: identifying relevant studies

#### Criteria for inclusion and exclusion of studies

The population of interest in this review consists of perioperative adult patients who have undergone a non-cardiac (including vascular) surgery or procedure. Given that the impact of sex and gender on outcomes in cardiac surgery is related to distinct anatomical and pathophysiological mechanisms that were recently addressed in systematic reviews and a meta-analysis, cardiac surgery and venous thromboembolic disease will be excluded from this review [24, 25]. Considering that we are investigating the role of sex and gender in the literature, the population will not be restricted based on either sex or gender.

Focusing on entities associated with atherosclerotic pathophysiology, the review will draw on the Standardized Endpoints in Perioperative Medicine (StEP) initiative validated outcomes of interest to include cardiovascular death, major adverse cardiovascular event (MACE), myocardial infarction (MI), stroke, cerebrovascular accident (CVA), heart failure (HF), arrhythmia, myocardial injury after non-cardiac surgery (MINS), and non-fatal cardiac arrest [26].

The perioperative period can pertain to the timing of either exposure (for example intraoperative hemorrhage) or the outcome, or both. The perioperative period is defined as the timeframe surrounding a surgical procedure, meaning the days and weeks leading up to and within 30 days after the surgical procedure [27].

Studies published from January 1st, 2010, to the present date will be considered for screening. The rationale for this time frame is to capture the impact of policies and recommendations for the inclusion of sex and gender considerations by major national and international regulatory and funding agencies [10].

Study types that will be considered in screening include clinical trials and observational studies. Systematic reviews, narrative reviews, meta-analyses, general reviews, and protocols will be excluded.

A summary of the inclusion and exclusion criteria for this review can be found in Table 1.

**Table 1:** Eligibility criteria for inclusion in the review

	**Inclusion criteria**	**Exclusion criteria**
**Publication dates**	January 1, 2010, and more recent	Prior to January 1, 2010
**Language**	English Language	Non-English
**Participants/population**	Perioperative adult (within 30 days of non-cardiac surgery)	Perioperative pediatric/neonatal
**Setting**	All non-cardiac surgeries/procedures	Cardiac surgeries involving cardiopulmonary bypass
**Exposure(s)**	Surgery, cardiovascular or other comorbidity, biomarker or other exposure	Congenital heart disease
**Outcomes(s)**	Cardiovascular death, MACE, MI, MINS, CVA/stroke, HF, arryhthmia, non-fatal cardiac arrest (on their own or > 1/2 of outcomes in composite).	VTE, PE
**Sources**	Clinical trials and observational studies (cohort, case-controlled, cross-sectional)	Case reports, case series, protocols, systematic reviews, narrative reviews, metanalyses, protocols or other secondary research. Studies with < 100 participants

Table abbreviations: *MACE* Major adverse cardiovascular event, *MI* Myocardial infarction, *CVA* Cerebrovascular accident, *HF* Heart failure, arryhthmia, *MINS* Myocardial injury after non-cardiac surgery, *VTE* Venous thromboembolism, *PE* Pulmonary embolism

#### Search strategy

The search strategy was developed by examining key words and index terms in a sample of relevant articles. Four main concepts, “perioperative,” “cardiovascular outcomes,” “sex and gender,” and “epidemiology” were identified. Keywords, phrases, and subject headings related to each concept were identified. Details of the search strategy are provided in Appendix 1 and an example of a search (PubMed) in Appendix 2. A medical librarian was consulted to assist with the selection of databases, development of search strategy, and its implementation in identifying peer-reviewed publications relevant to the objectives.

The search will include two databases (Medline, Embase) and the Cochrane Central Register of Controlled Trials. The databases were chosen for the most comprehensive major journal collections and detailed options for the conduct of complex searches. The search strategy is broad and should capture the majority of articles of interest. Reference lists of articles will be manually searched to find additional related articles to include for screening.

We expect to find a considerable proportion of studies to be relevant on 1st screening. Search results will be validated by benchmarking the proportion of relevant studies to 20%. After sorting search results by date of publication, the most recent 100 and the most remote 100 studies will be screened and the proportion of relevant titles noted. If the proportion is higher than 30% or lower than 10%, the results will be examined to decide which search terms may have to be less restrictive or more specific. In addition, 10 relevant “must include” studies known to authors will be used as markers to ensure that the search is appropriately calibrated.

The search results will be imported into Covidence (Covidence systematic review software, Veritas Health Innovation, Melbourne, Australia. Available at https://www.covidence.org).

### Stage 3: study selection

Once the results are imported into Covidence and deduplicated, titles and abstracts will be screened by two independent reviewers based on the pre-specified inclusion and exclusion criteria. When conflicts arise, a third reviewer will be consulted if consensus cannot be reached. To calibrate inclusion/exclusion criteria and instructions to reviewers, an interim assessment of the level of agreement will be conducted once around 10% of the studies have been screened by both reviewers. If a high level of agreement (> 90%) has not been achieved, the research team will discuss the reasons in detail and review the inclusion criteria with the possibility of revision and clarification. This process will be repeated after any revision and reported in the final review [28].

After the initial screening, selected articles will be retrieved in full-text form and assessed against inclusion and exclusion criteria. Exclusion of studies and the rationale will be documented at every stage of the process. The process and outcomes of the final and complete selection of studies to be included in the review will be documented and reported in a Preferred Reporting Items for Systematic Reviews and Meta-analyses extension for scoping review (PRISMA-ScR) flow diagram.

### Stage 4: charting the data

Only the data relevant to the research questions will be extracted from the final articles selected for full-text review. Extraction will be completed by two independent reviewers using the standardized data extraction tool developed in concordance with the data from JBI guidance for scoping reviews and set-up in Covidence by the research team [29]. The preliminary data extraction tool can be found in Appendix 3. We anticipate that all components of articles will have to be accessed for data extraction (discussion, tables, supplementary materials, etc.). The data extraction process will be piloted using a sample of studies (3–4 articles selected for full-text review from each type of evidence source) in order to improve and further standardize the process. Refinement of the data extraction tool will thus follow iteratively, whereby the team will pilot and then address issues such as missing or redundant items, the need for further clarification, and deviations from the anticipated time frame for extraction.

Quality assessment of data extraction will be performed once 50% and 100% of the chosen studies have been processed by pulling a random sample of 10% and verifying the extracted information. Any deviations from the process will be analyzed for root causes and addressed in team meetings.

Regular team meetings will be conducted to support the process. A log highlighting team communications and actions will be reported in a separate document in order to maintain transparency of reporting and results.

This work is not considered formal knowledge synthesis of findings on specific differences between the sex and gender categories. Critical appraisal (risk of bias and research quality assessments) is typically not involved in scoping review methodology and will, therefore, not be conducted.

### Stage 5: collating, summarizing, and reporting of results

A summary of the results will be reported using the PRISMA-ScR guidelines [23].

The extracted information will be organized and analyzed to show the study demographics (author, year, journal, number of citations, country), the design and context of each study, and where and how sex and gender are reported in the article.

Descriptive statistics will be used to summarize the review findings illustrated using tables and graphs. The overall proportion of papers with sex/gender reporting (and the categories of methodologic purpose) will be presented as the primary outcome. Temporal trends of proportions will be drawn (Cochrane-Armitage test) and results stratified by authors’ specialty, study size, proportion of female/women participants, major theme, and methodology. Uni- and multi-dimensional graphing approaches will be used to visually convey the results.

A narrative summary of sex/gender reporting and major sex differences will be presented by each research theme. Major sex differences and gaps in research will be broadly discussed and highlighted (to the exclusion of effect sizes, variances, statistical tests, or other outcome pooling). Further opportunities for systematic reviews and meta-analysis will be identified for specific areas where the body of identified literature is deemed sufficient to support formal knowledge synthesis.

## Conclusion

This scoping review will present an overview of the sex and gender-specific research in perioperative cardiovascular medicine and report of the use of sex and gender from year 2010 to present time. Additionally, through this scoping review, areas of strength and knowledge gaps will be highlighted in order to inform future research projects. Our findings may serve to assess the effectiveness of the last decade of policies directed towards increasing sex and gender inclusion in medical research specific to perioperative cardiovascular complications.

## Data Availability

The preliminary search string and results generated in the development of this protocol are available from the corresponding author on reasonable request.

## Appendix 1

Search strategy:

1. Using Boolean operator “OR”, combine all terms within each concept.
2. Combine concepts using operator “AND”.
3. Set limits
  - Human, English, 2010+ publication date
  - Not review (systematic, metanalyses, narrative)

**Table:**

Search strategy example (PubMed)	
1	Perioperative* OR “peri-operative*” OR Postop* OR “post-op*” OR Perioperative care [MeSH Major Topic] OR Perioperative period [MeSH Major Topic] OR Postoperative complications [MeSH Major Topic] OR Postoperative Period [MeSH Major Topic] OR Postoperative care [MeSH Major Topic] OR Perioperative Medicine [MeSH Major Topic].
2	Cardiovascular* OR “myocardial infarction*” OR “heart attack*” OR Stroke* OR “heart failure” OR Arrhythmia* OR “Atrial fibrillation” OR “Diastolic dysfunction” OR “Cardiac arrest” OR Troponin* OR “natriuretic peptide*” OR Echocardiogra* OR Angiogra* OR Electrocardiog* OR Heart Diseases [MeSH Major Topic] OR Vascular Diseases [MeSH Major Topic] OR Cardiovascular system [MeSH Major Topic] OR Stroke [MeSH Major Topic] OR Troponin [MeSH Major Topic] OR Natriuretic peptides [MeSH Major Topic] OR Echocardiography ([MeSH Major Topic] OR Electrocardiography [MeSH Major Topic] OR Angiography [MeSH Major Topic].
3	“risk factor*” OR Epidemiolog* OR Incidence* OR Occurrence* OR Prevalen* OR Symptom* OR Prevent* OR Treat* OR Outcome* OR Risk factors [MeSH Major Topic] OR Risk Assessment [MeSH Major Topic] OR Epidemiology [MeSH Major Topic] OR Epidemiology [MeSH Subheading] OR Prevalence [MeSH Major Topic] OR Incidence [MeSH Major Topic] OR Signs and Symptoms [MeSH Major Topic] OR Prevention and control [MeSH Subheading] OR Therapeutics [MeSH Major Topic] OR Outcome Assessment, Health Care [MeSH Major Topic].
4	Sex OR Sexes OR Female* OR Women* OR Woman* OR Gender* OR Women(Mesh) OR Female (Mesh) OR Sex distribution (MesH) OR Sex characteristics (Mesh)
5	Noncardiac* OR “non-cardiac” OR MINS OR Myocardial injury OR noncardiac surgery.
6	1 AND 2 AND 3 AND 4 AND 5
7	LIMIT to 2010-2023, human, English

**Table:**

Sex- and gender-based reporting review: search concepts with associated terms				
#1 Period Population	Event #2 Cardiovascular	#3 Risk factors etc.	#4 Gender	#5 Noncardiac*
Perioperative*	Cardiovascular*	“risk factor*”	Sex	Noncardiac*
“peri-operative*”	“myocardial infarction*”	Epidemiolog*	Sexes	“non-cardiac”
Postop*	“heart attack*”	Incidence*	Female*	MINS
“post-op*”	Stroke*	Occurrence*	Women*	Myocardial injury noncardiac surgery
	“heart failure”	Prevalen*	Woman*	
	Arrhythmia*	Symptom*	Gender*	
	“Atrial fibrillation”	Prevent*		
	“Diastolic dysfunction”	Treat*		
	“Cardiac arrest”	Outcome*		
	Troponin*			
	“natriuretic peptide*”			
	Echocardiogra*			
	Angiogra*			
	Electrocardiog*			
Perioperative care (Mesh)	Heart Diseases (Mesh)	Risk factors (Mesh)	Women(Mesh)	
Perioperative period (Mesh)	Vascular Diseases (MesH)	Risk Assessment (Mesh)	Female (Mesh)	
		Epidemiology (Mesh)	Sex distribution (MesH)	
Postoperative complications (Mesh)	Cardiovascular system (Mesh)	Epidemiology (Mesh subheading)	Sex characteristics (Mesh)	
Postoperative Period (Mesh)	Stroke (Mesh)	Prevalence (Mesh)		
Postoperative care (Mesh)	Troponin (Mesh)	Incidence (Mesh)		
Perioperative Medicine (Mesh)	Natriuretic peptides (Mesh)	Signs and Symptoms (Mesh)		
	Echocardiography (Mesh)	Prevention and control (Mesh Subheading)		
	Electrocardiography (Mesh)	Therapeutics (Mesh)		
	Angiography (Mesh)	Outcome Assessment, Health Care (Mesh)		

## Appendix 2

Search sample (PubMed)

An example of a search (PubMed)

Date: August 14, 2023

**Table:**

Search	Query	Results
#9	Search: #1 AND #2 AND #3 AND #4 AND #5 Filters: Humans, English, from 2010 - 2023 Sort by: Most Recent	1151
#8	Search: #1 AND #2 AND #3 AND #4 AND #5 Filters: Humans, from 2010 - 2023 Sort by: Most Recent	1192
#7	Search: #1 AND #2 AND #3 AND #4 AND #5 Filters: from 2010 - 2023 Sort by: Most Recent	1232
#6	Search: #1 AND #2 AND #3 AND #4 AND #5 Sort by: Most Recent	2120
#5	Search: (noncardiac*[Title/Abstract]) OR (“non-cardiac”[Title/Abstract]) OR (MINS[Title/Abstract]) OR (Myocardial injury noncardiac surgery[Title/Abstract]) - Saved search Sort by: Most Recent	19,440
#4	Search: (sex[Title/Abstract]) OR (sexes[Title/Abstract]) OR (Female*[Title/Abstract]) OR (Women*[Title/Abstract]) OR (Gender*[Title/Abstract]) OR ((((“Women”[Mesh]) OR “Female”[Mesh]) OR “Sex Distribution”[Mesh]) OR “Sex Characteristics”[Mesh]) - Saved search Sort by: Most Recent	10,257,688
#3	Search: (“risk factor*”[Title/Abstract]) OR (Epidemiolog*[Title/Abstract]) OR (Incidence*[Title/Abstract]) OR (Occurrence*[Title/Abstract]) OR (Prevalen*[Title/Abstract]) OR (Symptom*[Title/Abstract]) OR (Prevent*[Title/Abstract]) OR (Treat*[Title/Abstract]) OR (Outcome*[Title/Abstract]) OR ((((((((((“Risk Factors”[Mesh]) OR “Risk Assessment”[Mesh]) OR “Epidemiology”[Mesh]) OR “epidemiology” [Subheading]) OR “Prevalence”[Mesh]) OR “Incidence”[Mesh]) OR “Signs and Symptoms”[Mesh]) OR “prevention and control” [Subheading]) OR “Therapeutics”[Mesh]) OR “Outcome Assessment, Health Care”[Mesh]) - Saved search Sort by: Most Recent	16,191,386
#2	Search: (Cardiovascular*[Title/Abstract]) OR (“myocardial infarction*”[Title/Abstract]) OR (“heart attack*”[Title/Abstract]) OR (Stroke*[Title/Abstract]) OR (“heart failure”[Title/Abstract]) OR (Arrhythmia*[Title/Abstract]) OR (“Atrial fibrillation”[Title/Abstract]) OR (“Diastolic dysfunction”[Title/Abstract]) OR (“Cardiac arrest”[Title/Abstract]) OR (Troponin*[Title/Abstract]) OR (“natriuretic peptide*”[Title/Abstract]) OR (Echocardiogra*[Title/Abstract]) OR (Angiogra*[Title/Abstract]) OR (Electrocardiog*[Title/Abstract]) OR (((((((((“Heart Diseases”[Mesh]) OR “Vascular Diseases”[Mesh]) OR “Cardiovascular System”[Mesh]) OR “Stroke”[Mesh]) OR “Troponin”[Mesh]) OR “Natriuretic Peptides”[Mesh]) OR “Echocardiography”[Mesh]) OR “Electrocardiography”[Mesh]) OR “Angiography”[Mesh]) - Saved search Sort by: Most Recent	3,907,307
#1	Search: (((((perioperative*[Title/Abstract])) OR ((“peri-operative*”))) OR ((Postop*[Title/Abstract]))) OR ((“post-op*”[Title/Abstract]))) OR (((“Perioperative Period”[Mesh] OR “Perioperative Care”[Mesh] OR “Perioperative Medicine”[Mesh]) OR ( “Postoperative Period”[Mesh] OR “Postoperative Complications”[Mesh] OR “Postoperative Care”[Mesh] ))) - Saved search Sort by: Most Recent	1,316,686

## Appendix 3

Data extraction tool with guidance

**Table:**

**Variable**	**Label**	**Type**	**Values**	**Guidance**
**ID**	Study ID	Integer		
**Author**	Author’s last name	String		
**Journal**	Name of the source of publication	String		
**Year**	Year of publication	Num		
**Country**	Country of publication	String		
**Affiliation**	Primary author’s affiliation	String		Institutional or academic.
**Specialty**	Primary author’s specialty	Categorical	1 – cardiology 2 – medicine 3 – anesthesia 4 – epidemiology 5 - other	Primary author’s departmental affiliation or search online.
**Methodology**	Study design	Categorical	1 - clinical trial 2 - prospective cohort 3 - historical cohort 4 - case-control 5 - cross-sectional 6 – ecologic 7 - other	Stated in the title, abstract or methods. Select one.
**Size**	Total participant number	Integer		
**Women**	Percentage of female/women participants in the study	Continuous		Found in results under “demographics” or Table 1.
**Min age**	Minimum eligible age	Integer		Minimal intended age for study enrollment.
**Max age**	Maximum eligible age	Integer		Enter 1 if not specified.
**Mean age**	Mean age of study participants	Continuous		Found in demographic table.
**Outcomes**	Study outcomes	Categorical	1 – mortality 2 – MACE 3 – MINS 4 – MI 5 – readmission 6 – biomarkers (troponin, BNP) 7 – arrythmia 8 – CVA/stroke 9 – EFpHF (diastolic) 10 – EFrHF (systolic) 11 – RHF 12 – non-fatal cardiac arrest 13 - other	Multiple selections.
**Data**	Data availability	Categorical	0 – no 1 – available 2 – unclear	Is data available for secondary analysis? Look in data availability statement, usually before references.
**Definition**	Definition of sex/gender provided	Categorical	0 – no 1 – yes 2 – n/a	How are categories defined? Use n/a if study is not considering sex/gender.
**Binary**	The number of categories used for sex/gender	Categorical	1 – binary 2 – non-binary	Any addition of a third category is non-binary.
**Guideline**^**1**^	Guidance for appropriate methodological inclusion of sex/gender in research	Categorical	1 – none stated 2 – SAGER 3 – CIHR 4 – NIH 5 – journal 6 – other	
**Appropriateness **^**1**^	Appropriateness of use of terminology	Categorical	0 – no 1 – yes 2 – n/a	Appropriate: consistent use of male/female/intersex for sex; man/woman/gender-diverse for gender
**Interchangeability**^**1**^	Interchangeable use of terminology	Categorical	0 – no 1 – yes 2 – n/a	Noninterchangeable: consistent use of sex to describe biological attributes and gender for sociocultural attributes.
**Rationale**	Rationale for including (or not) sex/gender	Categorical	0 – no 1 – yes 2 – n/a	Is the rationale provided? Pick and answer regardless of whether the article mentions sex/gender.
**Reporting section**	Section of the article in which sex/gender are reported	Categorical	1 – title 2 –abstract 3 – keyword 4 – methods 5 – results 6 – discussion 7 – conclusion 8 – tables/graphs 9 – additional files 10 – not reported	Multiple selections.
**Role in methods**	The role of sex/gender in study methodology	Categorical	1 – pre-specified as an outcome 2 – pre-specified enrolment ratio or criteria 3 – to describe the population 4 – confounding variable 5 – stratifying variable 6 – unclear 7 – other 8 – article not including sex/gender in any way	Examples: Option—a study that aims to recruit equal number of each sex or uses sex stratified randomization. Option 3—demographic description or Table 1. Option 4—when outcome is “adjusted for” sex.
**Primary outcome**	Sex disaggregated primary outcome	Binary	0 – no 1 - yes	
**Stratified outcome**	Outcome variable that was stratified by sex/gender.	Categorical	1 – mortality 2 – MACE 3 – MINS 4 – MI 5 – readmission 6 – biomarkers (troponin, BNP) 7 – arrythmia 8 – CVA/stroke 9 – EFpHF (diastolic) 10 – EFrHF (systolic) 11 – RHF 12 – non-fatal cardiac arrest 13 – other 14 – not stratified by sex-gender	Multiple selections. Choose outcome(s) that were stratified by sex/gender.
**Aim**	Stated aim of the research.	Categorical	1 – risk factor analysis 2 – risk stratification 3 – prognostic value 4 – validation of predictor/test/model 5 – diagnostic score/symptoms 6 – prophylaxis 7 – treatment 8 – outcomes 9 – survival 10 – other 11 – unclear	Major purpose of the study whether related to sex/gender or not. Single selection.
**Summary**	Summary of findings			Use to describe sex/gender pertinent findings.
**Narrative**	Narrative description			Use if above fails to completely capture an aspect of the study that is relevant to sex/gender.
**Notes**				Team or admin notes.

^1^Gogovor A, Zomahoun HTV, Ekanmian G, Adisso ÉL, Deom Tardif A, Khadhraoui L, Rheault N, Moher D, Légaré F. Sex and gender considerations in reporting guidelines for health research: a systematic review. Biol Sex Differ. 2021 Nov 20;12(1):62. 10.1186/s13293-021-00404-0. PMID: 34801060; PMCID: PMC8605583

## Abbreviations

MINS: Myocardial injury following non-cardiac surgery
MI: Myocardial infarction
EFpHF: Heart failure with preserved ejection fraction
EFrHF: Heart failure with reduced ejection fraction
RHF: Right heart failure
MACE: Major adverse cardiovascular event
MI: Myocardial infarction
CVA: Cerebrovascular accident
BNP: Brain natriuretic peptide
MINS: Myocardial injury after non-cardiac surgery
SAGER: Sex and gender equality in research
CIHR: Canadian Institutes of Health Research
NIH: National Institutes of Health
